# Validation of a human-serum-based *in vitro* growth method for drug screening on juvenile development stages of *Schistosoma mansoni*

**DOI:** 10.1371/journal.pntd.0009313

**Published:** 2021-03-30

**Authors:** Valentin Buchter, Pierre H. H. Schneeberger, Jennifer Keiser

**Affiliations:** 1 Swiss Tropical and Public Health Institute, Basel, Switzerland; 2 University of Basel, Basel, Switzerland; Texas Biomedical Research institute, UNITED STATES

## Abstract

**Background:**

Schistosomiasis affects over 200 million people worldwide but only praziquantel is available for treatment and control. Drug discovery is often based on phenotypic drug screening, involving different parasite stages retrieved from infected mice. Aiming to reduce animal use, we validated an *in vitro* growth method for juvenile *Schistosoma mansoni* for the purpose of drug sensitivity assays.

**Methodology/Principal findings:**

We compared inter–batch variability of serum, worm size and organ development, gender distribution, and drug sensitivity between *in vitro* and *in vivo* grown worms over different life stages. *In vitro* developed *S*. *mansoni* in Hybridoma medium supplemented with 20% human serum were similar in size as *in vivo* worms until 28 days of incubation (males 1.4 ± 0.2 mm, females 1.1 ± 0.5 mm long). qPCR analysis revealed similar gender distribution both on newly transformed schistosomula and worms grown for 21 days. Worms developed *in vitro* and *in vivo* were similarly sensitive to praziquantel from 7 to 35 days of development with the exception of 21 days of development, where a slightly lower activity was observed for the *in vitro* grown worms (IC_50_: 0.54 μM *in vitro*, 0.14 μM *in vivo* 72 hours post-incubation). The evaluation of five additional drugs revealed a similar sensitivity on worms developed for 21 days, with the exception of mefloquine, where we observed a 10-fold lower sensitivity on *in vitro* developed schistosomes when compared to *in vivo* grown (IC_50_: 4.43 μM *in vitro*, 0.48 μM *in vivo*).

**Conclusion:**

A large number of juvenile *S*. *mansoni* worms can be grown *in vitro*, which show similar drug sensitivity, gender distribution, size and morphology as the worms recovered from rodents, supporting the use of this method in drug screening efforts.

## Author summary

Schistosomiasis is a water-borne disease affecting over 200 million people worldwide and praziquantel is the only drug available for treatment. Although this drug is effective on all *Schistosoma* species on the adult stage of development, its activity is only partial against early infection stages. In addition, this has been the only drug in use for over 40 years, a situation that poses selective pressure towards resistant worms and highlights the urgent need of new drugs. The discovery and development of new drugs is often based on drug screenings, performed on worms collected from infected mammals. In line with animal welfare recommendations and aiming to increase throughput and reduce the assay costs, we validated a recently established *in vitro* method to grow juvenile *S*. *mansoni* as a drug screening tool. The *in vitro* established worms have similar gender distribution, size and drug sensitivity as worms collected from mice.

## Introduction

Schistosomiasis is a human acute and chronic parasitic disease caused by five different trematode species (*Schistosoma haematobium*, *S*. *intercalatum*, *S*. *japonicum*, *S*. *mansoni and S*. *mekongi)* that affected over 229 million people in 2018, in 78 countries [1]. Despite all efforts to control the disease, which include large-scale population treatment with the only available drug praziquantel [1], the disease remains a significant public health problem [1,2]. Although drug resistance has not yet been documented in human medicine, treatment failures have been observed [3], and resistance to praziquantel could readily be induced in the laboratory [4]. Considering the high selective pressure due to a continuous and unique use of praziquantel in the treatment of schistosomiasis, and the fact that the efficacy of praziquantel is limited and inconsistent against immature worms [3], the development of alternative broad spectrum drugs is an urgent need [3].

One of the strategies used in drug discovery and development for schistosomiasis is the screening of drug libraries on different parasite stages *in vitro*. While drug screening on the larval stage (newly transformed schistosomula, NTS) [5,6] can be performed fully *in vitro*, infected mice or hamster are needed, when compounds need to be tested on juvenile and adult worms. Working with the juvenile stages of the parasites is particularly cumbersome, since the collection of worms by blood perfusion is a time intense process with low recovery rates and high costs. Moreover, because only few worms per animal can be collected, the use of juvenile worms collected from rodents is not in line with the 3R principle (reduce, replace, refine) for animal welfare in science [5,7].

Hence, a reliable and robust *in vitro* growing procedure for juvenile *S*. *mansoni* generating a large number of worms is required. During the 1960 and 70’s Clegg [8] and Basch [9] developed techniques for *in vitro* development for *S*. *mansoni*, that are still used today [10,11]. These methods rely on the addition of red blood cells (RBC) [8,9] or complex culture media and are therefore subject to high variability, are time consuming and due to the presence of the red blood cells in the medium, the phenotypic microscopy readout becomes imprecise [12,13]. Additionally, the success rate of development is as low as 10% of the original number of larvae incubated [14], the size of the worms is 20 to 40% smaller, and development is slower when compared to the *in vivo* counterparts [9]. Frahm *et al*. recently described a novel, cell-free method to grow *S*. *mansoni* cercariae into juvenile worm stages, thus decreasing the hurdles associated with previous methods [13]. However, a systematic comparison of developmental bias between this method and *in vivo* grown schistosomes, which is considered the gold standard [15], has not been conducted to date.

The aim of our study was to evaluate the reproducibility of a cell-free method to grow juvenile *S*. *mansoni* [13] and to validate its applicability for drug screening assays, comparing the assay results with worms developed *in vivo*. We evaluated the parasites’ development in medium containing serum of five different human donors individually, and as a pool of sera. Next, we compared the size, organ development and morphology of the worms developed i*n vitro* and *in vivo*. To avoid gender bias in the drug assays, we evaluated by means of qPCR and microscopy, the gender of the NTS which gave origin to the juvenile worms, the gender of developed worms as well as the gender of non-developing worms. Finally, to evaluate the applicability of this method for drug screening purposes, we quantitatively compared the sensitivity of the *in vitro* developed worms to six different drugs, with the sensitivity of the worms developed *in vivo*.

## Methods

### Ethics statement

All animal experiments were authorized by the Canton Basel Stadt, Switzerland (license number 2070).

### Parasites

The life cycle of *S*. *mansoni* is established at Swiss TPH [5]. Briefly, *Biomphalaria glabrata* (Egypt) snails were infected with 6–8 *S*. *mansoni–*Liberian Strain miracidia and 6 weeks after infection, the cercariae shed from the snails were used for animal infection or mechanically transformed to newly transformed schistosomula (NTS) [5] and used for *in vitro* growing.

### Human serum

Human serum (HSe) was collected at the blood donation center in Basel, Switzerland, from five anonymous and randomly chosen donors. Parasite growth was tested with each serum sample, individually, or with a pool of all sera, composed by the mixture of equal parts of each of these sera. The effect of heat inactivation of the complement proteins in the serum was evaluated. Two batches of developing worms were incubated with heat inactivated serum and two with normal serum. Inactivation was achieved by placing serum samples in a 56°C water bath for 30 minutes.

### *In vitro* growing assay

For the *in vitro* growth procedure, 30 to 50 NTS were placed into each well of 96 well transparent polystyrene flat bottom plates containing 100 μl medium. We compared the growth of the schistosomes in both Dulbecco’s modified eagle’s medium (DMEM, Sigma-Aldrich, Buchs, Switzerland) and HybridoMed (HM) DIF1000 serum-free medium (VWR international, Dietikon, Switzerland) both supplemented with 20% HSe and 500 U/mL penicillin, 500 mg/mL streptomycin (LuBioScience, Switzerland) [13]. The plates were kept in the incubator with 5% CO_2_ and at 37°C. To avoid evaporation and keep humidity and temperature constant, the outer rows of the plate were filled with culture medium, but no NTS were added. The medium was changed twice a week until use. Pictures of the worms were taken weekly, using a Canon photo camera EOS digital mounted on a Carl Zeiss Primovert microscope and using 200x, 100x and 40x magnifications.

We compared the schistosome development in five different batches of sera and a pool of these sera. We incubated two wells with each of the sera, and allowed the worms to develop for 21 days. After this period we took pictures of the wells and counted the number of dead and alive worms in each well and the number of worms which had reached the organogeny stage (Table 1). The incubation with the pool of sera was repeated once. For subsequent experiments this batch of pooled sera was used.

**Table 1:** Assignment of developmental stage according to morphology and organ formation.

Stage		Time in mouse model (d)	Description
Lung stage	Larvae	7–8	Elongated schistosomula, without specific gender differentiation and increased movement. Suckers are hardly visible and there is no evidence of digestive system formation.
Gut development stage	Juvenile	15	Considerable increase in volume takes place and the gut ceca turns visible and united behind the ventral sucker. Males, thicker and longer, can be differentiated from females, which are thinner and still elongated. While in males the ventral and oral sucker are clearly evidenced, this is not the case for females. Defined as liver stage by Frahm *et al*. [13]
Organogeny stage	Juvenile	21	Growing continues in length and the gut ceca elongates. Male schistosomes start to develop the lateral extensions of the body and two testicles are visible.
Gametogeny stage	Juvenile	28	Males develop 8 testes while females have a small ovary. Mating occurs for the first time and the females are enclosed by the lateral extensions of the males’ body.
Adult stage	Adult	35–55	First eggs from *in vivo* developed *S*. *mansoni* are produced 34–35 days after infection. Despite highly variable, the size of females ranges from 5.1 to 9.4 mm and males from 5.0 to 8.8 mm. The vitellaria are well developed. Spermatozoa can be seen in all 8 testes of the male, and a seminal vesicle is present. By day 55 day post infection all worms have matured. While the tegument of females is smooth, the tegument of males is covered by spines.

### *In vivo* developed worms

Three-week-old female NMRI mice were ordered from Charles River (Sulzfeld, Germany) and were allowed to acclimatize to the new environment for one week before being manipulated. After this time, the mice were infected subcutaneously with 1000 cercariae for the 7 and 14 day time point and with 300 cercariae for the remaining time points, following a procedure described elsewhere [5]. Seven, 14, 21, 28 and 35 days after infection, the mice were euthanized and the worms were collected by blood perfusion. The perfusion solution (PS) consisted of 8.5 g NaCl and 7.5 g sodium citrate (Sigma Aldrich, Buchs, Switzerland) per liter of distilled water. The PS was sterile filtered and used at 4°C. For euthanasia, we injected a solution containing 87 μl of Esconarkon (sodium pentobarbital 300 mg/ml, Streuli Pharma, Uznach, Switzerland) and 13 μl of sodium heparin at 5.000 U.I./ml(Braun Medical AG, Sempach, Switzerland) subcutaneously in the third quadrant of the abdomen. Cervical dislocation was performed as second killing method, in line with the Swiss animal regulations. Afterwards, an incision in the abdomen was done and extended to the chest, removing the ribs, yet taking care not to damage the blood vessels.

The seven day-old worms were collected from the lungs as follows: the descendant thoracic portion of the aorta was cut with a scissor, to allow the blood to flow out by heart pressure. Later, 10 to 20 ml PS supplemented with 10 units heparin/ml were injected into the right ventricle of the heart to flush the blood from the lungs. The blood was discarded. The lungs were further removed from the chest, placed in glass beakers with 1–2 ml culture medium and minced into small pieces, transferred in 50 ml falcon tubes, filled with culture medium and incubated for 3 hours at 37°C and 5% CO_2_ to allow the worms to leave the tissue. Afterwards, the suspension was filtered through a 30-mesh screen and the filtrate was centrifuged 3 min at 100 g. The supernatant was removed and replaced with 10 ml fresh medium and transferred to a 24 well plate. The well plate was kept in the incubator at 37°C and 5% CO_2_ until use for the assays within 24 hours. For drug sensitivity assays the worms were collected using a 10 μl pipet.

For the 14 day-old worms and later development time points, we applied the following protocol: An incision was made in the portal vein and the blood was allowed to flow into a cylindrical glass container (23 cm diam. 3500 ml, Cat No.AYH0.1, Roth AG, Switzerland) placed below the mouse. The PS was used to rinse the area around the incision in the portal vein and thus prevent worms adhering to abdominal viscera. Next, 20 ml of PS were injected into the cava vein to retro-perfuse the liver and remove the worms contained in this organ. The full perfusate fluid from the glass container was collected into 50 ml falcon tubes and centrifuged for 3 minutes by 100 g. Next, the supernatant was removed and replaced with fresh PS. The centrifugation and clean up procedure was repeated three times until a transparent fluid was obtained. Finally, the PS was replaced by 10 ml culture medium (HM + HSe + Pen/Strep) and the worms placed in 24 well plates. Worms were kept in these plates in the incubator at 37°C and 5% CO_2_until use, within 24 hours after collection. At the 14 days p.i. time point, additional worms harbored in the lungs were collected following the procedure described for the 7 day p.i. perfusion.

### Assessment of development

To evaluate the development of the worms grown *in vitro* or *in vivo* we adapted the description of Clegg [8] for *S*. *mansoni* developed in mice. In brief, we evaluated growth (increase in size), development (organ formation and tissue differentiation) and maturation (sexual organ formation) as summarized in Table 1.

### Size determination

For the calculation of the parasite’s size we used the software Fiji–Image J [16]. Pictures with 100x and 200x magnification were taken after 0, 7, 14, 21, 28, 35 days of *in vitro* development and the length of 11 males and females was measured per time point. From the worms developed *in vivo*, we measured the size at days 14, 21, 28, 35 as well as at day 49 post infection.

### Gender determination

DNA from individual worms was extracted using DNeasy PowerSoil Pro Kit (Qiagen, Hilden, Germany) extraction kits. We extracted DNA from NTS, 21 and 28 day old juvenile *S*. *mansoni* grown *in vitro*, both developed and undeveloped as determined by morphological assessment, and matured adult worms which were grown *in vivo*.

We used a qPCR assay described by Chevalier *et al*. [17] to unambiguously determine the gender of the worms at each developmental stage. Briefly, this qPCR assay was designed to target two gender-specific regions of the chromosomal DNA, namely region W6 for females, and region Z for males. Accordingly, samples were classified as females if positive solely for region W6, and as males if positive only for region Z. For samples amplifying both regions, we measured the cycle threshold difference between both amplification targets (Δ Cq = Cq_W6_ –Cq_Z_), allowing for the unambiguous classification as females (high W6 / low Z) or males (high Z / low W6).

To amplify the Z marker we used the primers described elsewhere [17] Smp 011570.1 (Eurofins genomics, Ebersberg, Germany, sequence F (5’–3’ orientation): TGGATGTTGGATAAGCTGGG, sequence R: TGGATCTGGATATCGAATGGTC) while for the W6 marker we used the primer Sm_W6 (Eurofins genomics, Ebersberg, Germany, sequence F: TGTGAAGCAAAGTGTTCACTG, sequence R: TTCATCAAGTCAATCACAGCTC) used at a final concentration of 0.3 μM for each reaction. Each reaction also contained 2 μl of 5x HOT FIREPol Eva Green qPCR Supermix (Solis BioDyne, Tartu, Estonia), 5.4 μl DNA free water (Thermo Scientific, Allschwill, Switzerland), and 2 μl DNA template for a reaction volume of 10 μl. The DNA was amplified with the following program: 95°C for 12 min, [95°C for 15 s, 60°C for 1 min] × 40 cycles using a Bio-Rad CFX96 qPCR instrument (Bio-Rad Hungary Ltd., Budapest, Hungary). A melting curve (60–95°C) at the end of the program was added to check specificity of amplification. qPCR data analysis was performed with the software CFX Maestro version 4.0 (Bio-Rad, Hercules, California, USA).

### Drug sensitivity assays and IC_50_ calculations

We evaluated the viability of the worms after 24, 48, and 72 hours of continuous drug exposure by means of visual microscopy. The worms’ viability was evaluated assigning a score ranging from 0 to 3, with 0.25 unit intervals. A score of 0 was given to dead worms, while a score of 3 was assigned to fully vital worms. The criteria used to score the viability were movement, frequency of body contractions, granularity and transparency and tegument morphology.

The activity of six drugs, namely auranofin (Enzo Life Sciences AG, Lausen, Switzerland), mefloquine (Sigma-Aldrich, Buchs, Switzerland), nicardipine (Sigma-Aldrich, Buchs, Switzerland), oxamniquine (kindly donated by Pfizer, NY, USA), oxethazaine (Sigma-Aldrich, Buchs, Switzerland) and praziquantel (Sigma-Aldrich, Buchs, Switzerland) was evaluated against both *in vitro* and *in vivo* developed *S*. *mansoni* by 21 days of development. For praziquantel, we additionally compared the drug sensitivity of worms developed for 7, 14, 28 and 35 days *in vitro* and *in vivo*. The concentrations used to calculate the IC_50_ values were: for praziquantel: 2.5, 1.25 and 0.625 μM, for oxamniquine: 50, 25, 12.5 μM, for auranofin, mefloquine, nicardipine and oxethazaine: 10, 5, 2.5, 1.25 μM. The selection of the dose range was based on previous published studies describing the sensitivity of different stages of development to these drugs [13,18–20]. The experiments were performed in duplicate using at least three worms per well and were repeated once. The drug effect was calculated by normalizing the viability of the worms exposed to the drugs, to the viability score of the control wells. The control wells consisted of worms incubated in the same culture media as the test conditions, and had the amount of DMSO corresponding to highest concentration of the assay (0.5% V/V). IC_50_ values were calculated using the software Graph Pad Prism V8.0 (San Diego, CA, USA).

### Statistical analysis

We analyzed the gender distribution of NTS and juvenile developed and undeveloped worms using binomial tests for dichotomous non-parametric comparisons in R, version 3.5.1 (2018-07-02) [21]. By PCR we investigated the gender of 18 randomly selected NTS provided by three different batches and 26 worms incubated *in vitro* for 21 days from which the gender could not be determined by microscopy (undeveloped worms), also provided by three different batches. For data interpretation, we applied the binomial test assigning a success to “female” and a fail to “male”. The null hypothesis was “the probability of success is equal to 0.5”. The null hypothesis was rejected if ρ<0.05.

Kruskal-Wallis tests were used to compare the length between *in vivo* and *in vitro* grown worms. In each case we compared the length of worms among the week development group. *P*-values were adjusted for multiple testing bias using the Bonferroni procedure. For comparison of the 35 days of development, we additionally performed a Student’s t-Test to focus on single comparisons of males and females *in vitro* versus *in vivo*.

## Results

### Assay establishment

NTS incubated in HM + 20% HSe showed weekly changes in morphology, bigger size and higher viability scores and movement than those incubated in DMEM + 20% HSe, in line with developmental phases described in the literature [8,13] (S1–S5 Figs). Based on these findings, we continued our study using HM medium + 20% HSe. The overall survival by means of this cultivation method was 99% within 21 days of development.

Subsequently we evaluated the reproducibility of the incubation conditions by testing five serum batches consisting of individual serum samples from different donors and a pool of all five sera mixed in equal parts. Although all individual batches of serum were able to promote the development of the parasites to further stages than the gut development stage (described as early liver stage by Frahm *et al*. [13]) after 21 days of incubation, the highest survival rate and rate of worms reaching the organogeny stage of development, was observed by the pool of sera (Table 2 and S6 Fig).

**Table 2:** Comparison of the promotion of development to the organogeny stage, by each of the sera added to HM.

Serum #	N	% survival	% developed organogeny
1	71	85.9 (0.3)	54.4 (1.6)
2	62	80.6 (0.9)	48.7 (15.9)
3	70	67.1 (7.1)	16.8 (2.5)
4	58	55.6 (9.9)	25.0 (3.2)
5	73	67.3 (3.0)	37.3 (20.6)
6	151	98.7 (1.3)	71.8 (9.4)

Serum 6 is the mixture of equal parts of sera 1 to 5.

The table shows the average and standard deviation (SD) of experimental duplicates by 21 days of *in vitro* incubation. The experiment on the pool of sera was repeated once.

Additionally, the complement system, tested by adding untreated or heat-inactivated serum, had no influence on the development of the parasites, judged by the size, survival rate and morphology of the parasites.

### Size comparison

**Fig 1:**
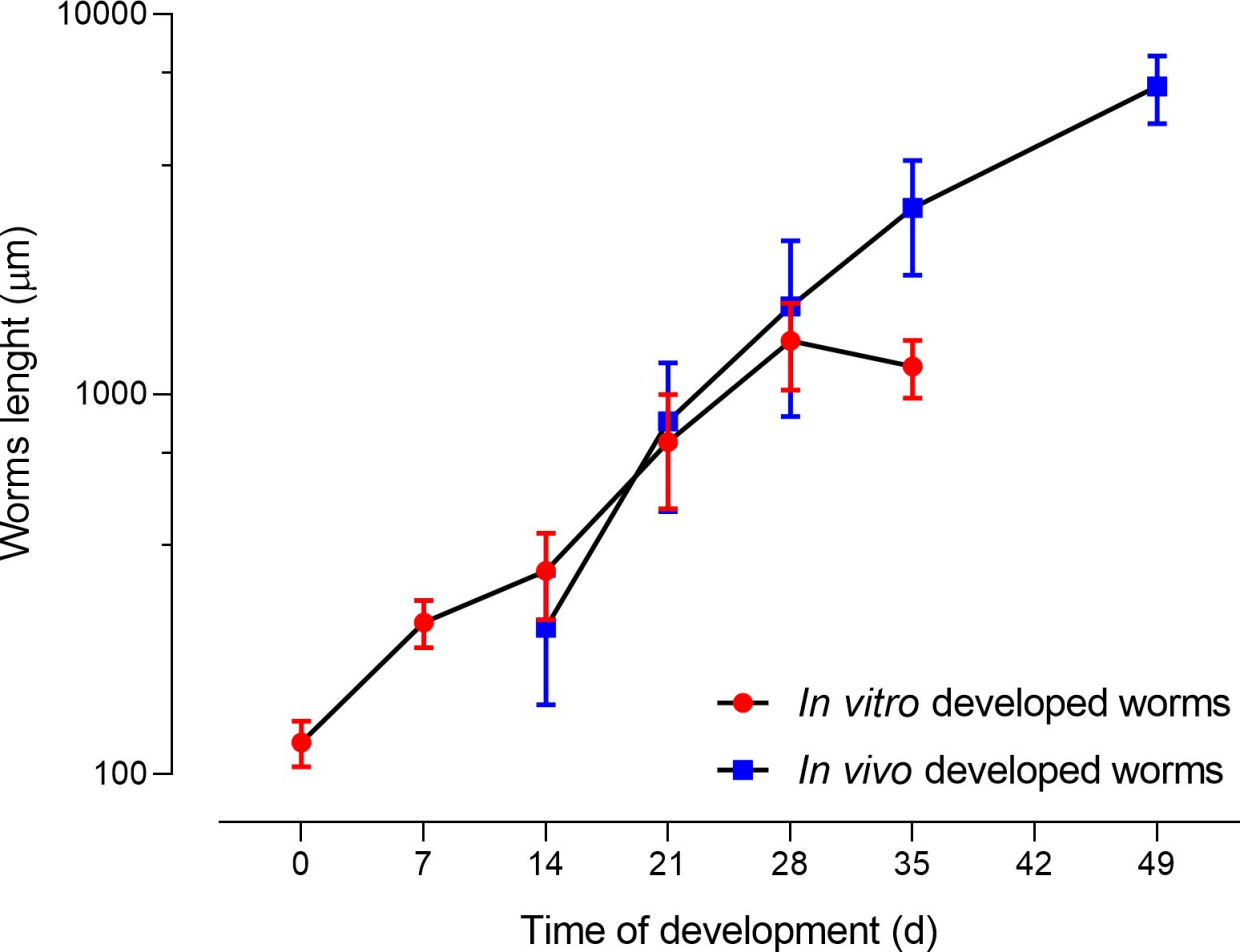
Comparison of the length of *S*. *mansoni* developed *in vitro* with those developed *in vivo*. Each point represents the average length of 22 worms including males and females.

We next evaluated the length of the *S*. *mansoni* grown *in vitro* and compared it to that of the worms developed *in vivo* (Fig 1). We found that the length of the worms developed *in vitro* correlated very well with the length of the worms developed *in vivo* for both genders until 28 days of development (S1 Table). After this time, the growth of the *in vitro* cultivated schistosomes reached a plateau and no further significant growth was detected, while the *in vivo* developed worms continued growing in length and reached sexual maturity. As a result, both males and females developed for 35 days *in vitro* were significantly shorter than those developed *in vivo* (ρ<0.01) (S7 Fig). For comparison, we also depict the adult worms retrieved from mice 49 days after infection, which had increased considerably in size (males: 7.2 ± 1.0 mm, females: 5.3 ± 0.9 mm).

### Gender determination

Under the described medium conditions, we were able to determine the gender by microscopy, from day 13 onwards and to keep the *in vitro* growing worms alive for at least 56 days. However, sexual maturity was not evident in any experiment and worms did not couple and therefore did not lay any eggs.

**Fig 2:**
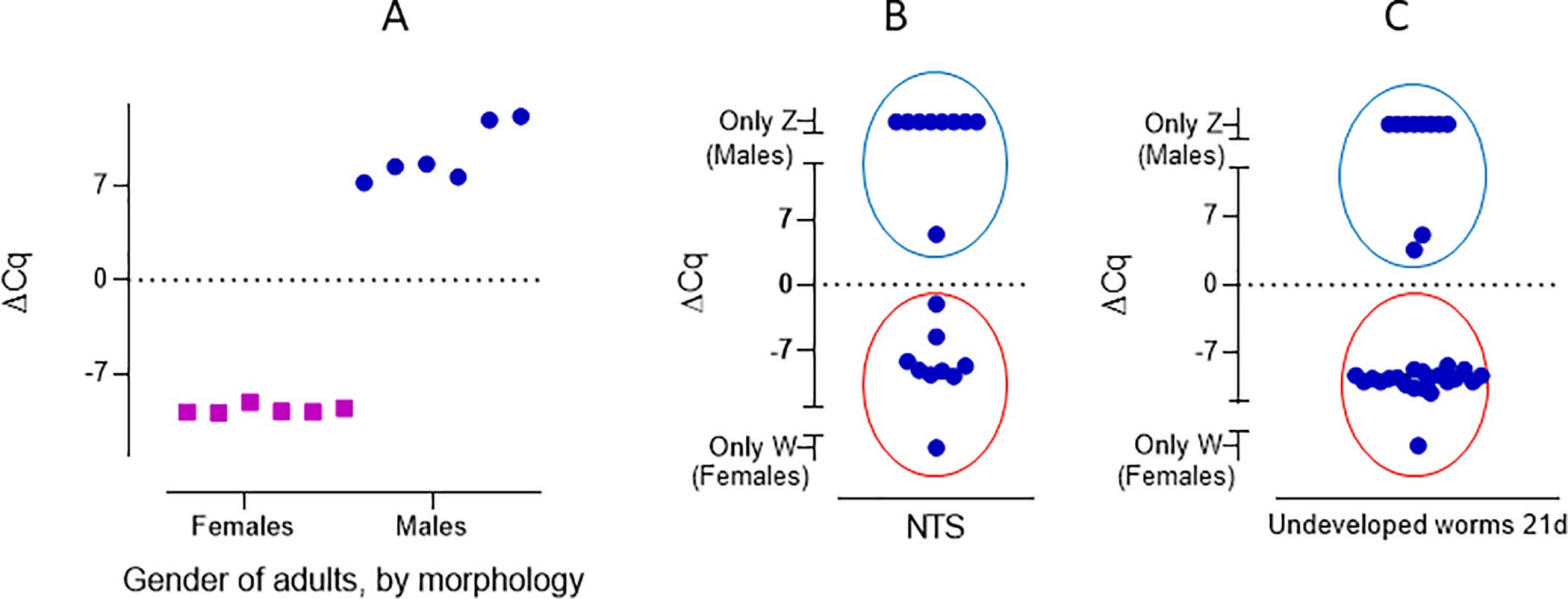
Comparison of the morphology and ΔCq of adult *S*. *mansoni* (A) NTS (B) and undeveloped worms by 21 days (C). A: adult worms, collected from mice 49 days post infection, classified as females (squares) and males (circles) by morphology, compared to ΔCq. B: gender distribution observed in NTS by means of qPCR. C: gender distribution of undeveloped worms, incubated for 21 days. The ΔCq for female worms was negative (red circle) and positive for males (blue circle), allowing unequivocal gender identification.

As described by Chevalier *et al*. [17], males present high levels of Z marker, while females present high expression of the W6 region. For worms expressing both markers, at any developmental stage, we calculated the Δ Cq (Cq _W6_ –Cq _Z_) value and found a difference of -9.21 ± 1.31 cycles for female *S*. *mansoni* and a positive Δ Cq for male worms (Fig 2), allowing us to unambiguously assign a gender to each worm. When considering the adult worms only, we observed that all samples amplified both markers. In males the ΔCq was 9.4 ± 2.2 (mean ± SD), while in females the ΔCq was -9.6 ± 0.3 (Fig 2A). We found a 100% agreement between morphology gender assignment and qPCR (Figs 2A and S8) when looking at a subset (n = 12) of adult worms (developed *in vivo*).

We were interested in identifying a possible bias in the gender distribution of the worms grown *in vitro*, which might influence results of the drug sensitivity assays. We first evaluated the gender distribution of the NTS by qPCR and found 50% of the parasites to be females (n = 18, *p* = 1) (Fig 2B). Subsequently, we evaluated the gender of parasites that were undeveloped by day 21 (worms that are not growing and for which no gender can be assigned by morphological assessment), and found that 65.4% of these were females. This difference was not significant (n = 26, *p* = 0.17) (Fig 2C). When considering the developed worms by 21 days of *in vitro* cultivation, by optical microscopy, no significant difference in the number of males compared to females was observed (males = 52%, *p* = 0.47, n_total_ = 193).

### Comparison of drug sensitivity

**Fig 3:**
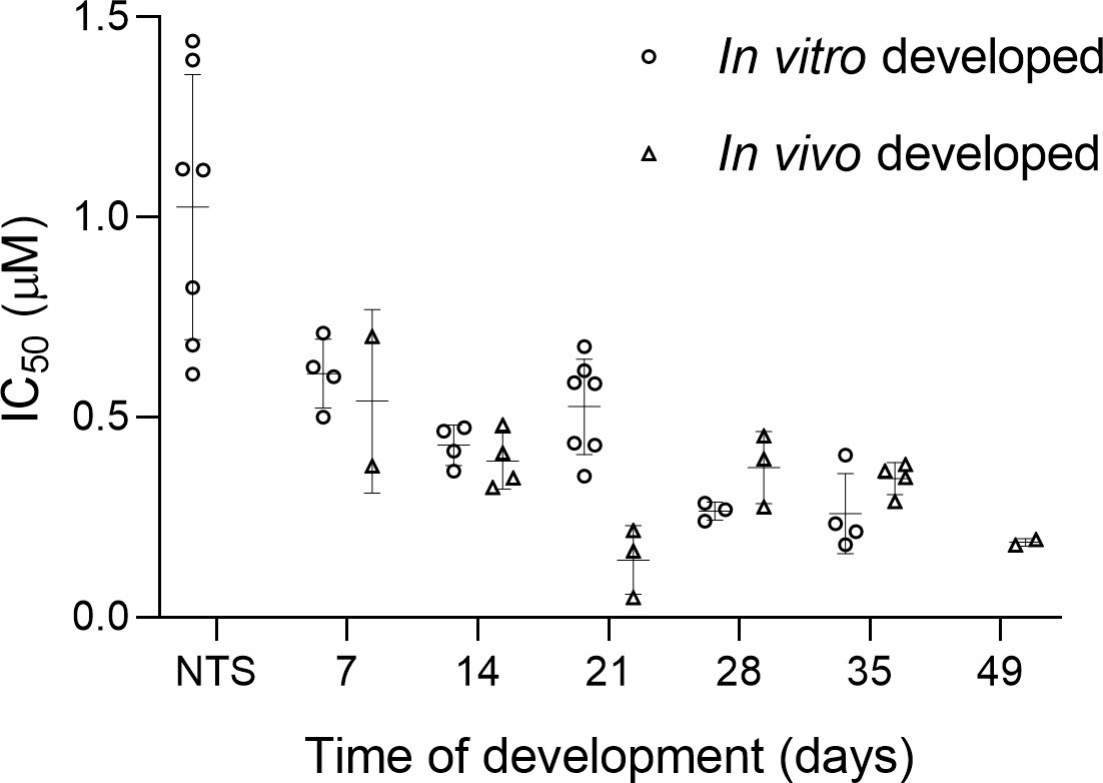
Sensitivity (IC_50_ values) of *S*. *mansoni* grown *in vivo* and developed *in vitro* exposed to praziquantel for 72 hours as a function of time. Each point in the figure represents an IC_50_ value, calculated from a full assay including four concentrations, three worms per concentration and conducted in duplicate.

We evaluated the activity of praziquantel on NTS and 7-to-35-day old *S*. *mansoni*, cultured *in vitro* and collected *in vivo*. As a reference, the activity of praziquantel on adult *S*. *mansoni* (grown *in vivo* for 49 days) was also evaluated (Fig 3).

Next, we exposed worms grown *in vitro* and *in vivo* to five additional marketed drugs with known antischistosomal activity and different mechanisms of action [20] and compared sensitivities after 21 days of development (Table 3). The drugs used were auranofin [22], mefloquine [23], nicardipine [20,24], oxamniquine [25] and oxethazaine [20,26]. Oxamniquine was not lethal against worms grown either *in vivo* or *in vitro*, resulting in IC_50_ values above the range of concentrations used. Despite considerable differences at early evaluation time points (24 and 48 hours) for auranofin, nicardipine and oxethazaine all drugs evaluated showed similar IC_50_ values at the 72 hours evaluation time. In contrast, we observed a different sensitivity among both growing methods, resulting in a 10-fold lower sensitivity to mefloquine for *in vitro* developed worms, compared to those developed *in vivo* at 72 hours post-incubation.

**Table 3:** Sensitivity (IC_50_ (SD)) comparison of *S*. *mansoni* incubated *in vitro* for 21 days compared to *S*. *mansoni* developed in mice for 21 days (μM).

		*In vitro*	*In vivo*
Auranofin	24 h	6.02 (12.35)	>100
	48 h	3.85 (3.64)	12.05 (6.30)
	72 h	3.93 (6.92)	5.31 (1.59)
Mefloquine	24 h	24.26 (0.00)	5.64 (0.79)
	48 h	9.31 (1.91)	11.67 (9.44)
	72 h	4.43 (0.20)	0.48 (0.20)
Oxamniquine	24 h	>100	>100
	48 h	>100	>100
	72 h	>100	>100
Praziquantel	24 h	0.74 (1.55)	0.54 (0.04)
	48 h	0.75 (0.71)	0.09 (0.09)
	72 h	0.54 (0.12)	0.14 (0.09)
Oxethazaine	24 h	>100	>100
	48 h	2.52 (0.14)	>100
	72 h	8.32 (10.32)	3.26 (3.23)
Nicardipine	24 h	6.87 (6.73)	12.45 (10.68)
	48 h	12.66 (20.48)	2.30 (3.24)
	72 h	2.09 (0.67)	2.05 (0.80)

## Discussion

Our aim was to validate the application of a method to grow *S*. *mansoni in vitro* [13] for drug sensitivity assays. We first evaluated the reproducibility of different batches of sera to promote the parasite growth, and once the method was established, we evaluated the length, gender distribution and drug sensitivity of the worms grown under *in vitro* conditions and compared them to *in vivo* collected worms.

As also described by Basch *et al*. [9], we found differences in worm development between sera provided by different donors, while complement inactivation did not influence worm development. The highest survival and development rates at 21 and 28 days of development were observed while using the pool of sera when compared to the serum of individual donors. In the previous study by Frahm *et al*. [13], serum provided by six different donors was evaluated, but inter-batch variability was not reported.

In mice, the full life cycle and an established infection takes from 5 to 8 weeks [5,8]. By this time in the mouse model, adults would have developed, coupled in the liver, moved to the mesenteric veins and the females started shedding eggs. We did not observe sexual maturity and pairing of the worms using the *in vitro* culture. This is a limitation when compared to the method described by Basch and colleagues, who managed to culture mature egg laying worms. However, these eggs were not viable, and the full life cycle could not be established until today [14,27,28]. In our study, no worms coupled even after 56 days of *in vitro* incubation, suggesting that sexual maturity is not only a matter of time, but more a factor of parasites/environment interaction, that triggers development, and future research could be oriented to identify these factors.

As known from previous studies [9], the development of schistosomes is subject to great variability [8] and we also observed it in worms grown both *in vitro* and *in vivo*. For example, in our experiments on 21-day old *in vivo* worms, 47.4% of the worms were not sufficiently developed to unambiguously assign a gender and there was also great variability in the parasites lengths (Figs 1 and S7 and S9). This finding is in line with the descriptions by Clegg *et al*. on *in vivo* grown juvenile *S*. *mansoni*, who also reported variability in the size of the worms, as well as in the time needed to progress from one stage to the next [8]. Although by 28 days of *in vivo* development we also found marked variability in their length (Fig 1), all worms could be morphologically classified either as males or females (S10 Fig), and a few worms had coupled.

The size determined for 7–14 day old worms grown *in vitro* are in line with findings described by Clegg *et al* [8]. For example, Clegg noted the size of the 7 day old (lung stage) worms to be 252 x 25 μm, similar to our study (*in vitro* grown worms: mean (SD): 251.1 (35.5) μm). However, contradictory findings were observed for the worms collected from day 28 to 49. In the aforementioned study, worms reached a maximum of 4.8 mm [8], which represented more than double the size of the largest worm (2.1 mm) observed in our study, after an incubation period of 28 days.

There is clear evidence of differential praziquantel sensitivity along the life cycle in infected animals [29–31] and humans [32], showing lower sensitivity to praziquantel in the juvenile stages of development. However, the differential sensitivity *in vitro* is under discussion [31,33]. Overall, studies agree that the highest sensitivity to praziquantel is observed in the adult stage [33,34] but the results on juveniles are diverse, showing reduced sensitivity until day 7 of development [13,29,35] or day 28 [33] and recovery of sensitivity by day 37 [33]. This was confirmed by our study as well, as we observed the highest activity of praziquantel on adults, low activity against NTS and moderate activity for the juvenile stages. The sensitivity to praziquantel along the life cycle was similar among the worms obtained by both growing methods, with the exception of the 21 days old stage, where we observed differences at the 48 and 72 hour IC_50_ values despite the similar sensitivity at the 24 hours evaluation time. The stage-specific sensitivity found in our studies *in vitro* is slightly different from studies evaluating the sensitivity to praziquantel in *S*. *mansoni* infected mice, where the lowest activity was observed against juvenile 28 day-old worms [36].

We also included oxamniquine as a negative control, since it is a pro-drug [25] that shows excellent clinical efficacy against chronic infections [32] and is also active on adult worms in *in vivo* models [37], but has limited activity against any of the developmental stages *in vitro* [38,39]. As expected, oxamniquine was inactive in both the *in vivo* and *in vitro* developed worms (IC_50_ > 100 μM).

We observed a slight difference in the sensitivity to mefloquine particularly at the 72 hour evaluation time point. The exact mechanism of action of mefloquine on schistosomes has not been elucidated yet. On *Plasmodium* spp. mefloquine is suggested to act via the inhibition of the hemoglobin detoxification mechanisms, causing an accumulation of toxic hem inside the parasite, leading to its death [40]. In *S*. *mansoni*, the same hemoglobin degradation products were found, suggesting a similar detoxification mechanism [41]. However, previous studies showed that although mefloquine was more active on *S*. *mansoni* in the presence of hemin [42], the drug was also active on the parasites in absence of blood or hemin itself [43], suggesting multiple mefloquine targets or mechanisms of action. Both our *in vitro* developed worms and those obtained by perfusion were incubated in blood-free medium; however, the worms collected from the mice still had blood remaining in the gut at the beginning of the assay, and this could explain the slightly higher activity of mefloquine on the *in vivo* worms compared to the ones grown *in vitro*, which have never had contact with hemoglobin. However, it is not clear why a difference in sensitivity to mefloquine was only observed 72 hours post-incubation. We also observed considerable difference in the activity of auranofin, oxethazaine and nicardipine, hinting to differences in the onset of action of the drugs between the *in vitro* and *in vivo* developed worms. Further studies are necessary to understand the underlying reasons for these results. Nonetheless, with exception of mefloquine, a good correlation between the assays was observed at the most commonly used, recommended 72 hours evaluation time point [5].

## Conclusion

We evaluated the size, gender, and drug susceptibility of juvenile *in vitro* developed *S*. *mansoni* and compared them to parasites grown *in vivo*. Overall, the size of the worms and gender was similar between both growing techniques until 28 days of development. Additionally, we observed similarity in sensitivity to auranofin, nicardipine, oxamniquine, oxethazaine and praziquantel, after 72 hours of incubation between the worms developed *in vitro* and the worms grown *in vivo* for 21 days. Despite the slightly different sensitivity to mefloquine at the 72-hour evaluation time point and considering the highly laborious method to collect juvenile *S*. *mansoni* from infected mice, this method is an excellent alternative to the development of juvenile *S*. *mansoni* in mice, as it allows the production of a much higher number of juvenile worms in an ethical manner and under very simple assay conditions, which can be used for drug screening on juvenile worms.

## Supporting information

S1 Table *S*. *mansoni* length (μm) according to time of development (in days) either *in vitro* or *in vivo*. (XLSX)

S1 Fig *S*. *mansoni* developed *in vitro* for 7 days. No gender identification possible. Amplification: A: 100x, B:200x. Scale bar: 200 μm. (TIF)

S2 Fig *S*. *mansoni* developed *in vitro* for 14 days By 14 days of development, the males are distinguishable from females. Amplification: A: 100x, B:200x. Scale bar: 200 μm.
(TIF)

S3 Fig *S*. *mansoni* developed *in vitro* for 21 days Amplification: 200x. U: undeveloped worm. Scale bar: 200 μm.
(TIF)

S4 Fig *S*. *mansoni* developed *in vitro* for 28 days. U: undeveloped worm. Amplification: A: 200x, B: 100x. Scale bar: 200 μm.
(TIF)

S5 Fig *S*. *mansoni* developed *in vitro* for 35 days. Amplification: 100x. Scale bar: 200 μm.
(TIF)

S6 Fig Comparison of five individual batches of sera and the pool of all of them. Comparison of the development of *in vitro* grown *S*. *mansoni* using HM + 20% HSe from five different donors by 21 days of development. Serum 6 is the combination of the other five sera mixed together in equal parts. Scale bar: 200 μm.
(TIF)

S7 Fig Comparison of the length of worms grown by both methods as a function of time, split by gender. By means of either growing technique the worms’ gender is only to be identified after 10–13 days of development. Each point represents one worm. Abbreviations: ND: not done, undef: undefined: the gender of the worms cannot be determined by optical microscopy.
(TIF)

S8 Fig Adult couples of *S*. *mansoni* developed *in vivo* for 49 days and incubated free of drugs for 24 hours. Amplification: 40x. Scale bar: 1 mm.
(TIF)

S9 Fig *S*. *mansoni* developed *in vivo* for 21 days A: male and two undeveloped worms 100X, B: undeveloped worms100X. U: undeveloped worm. Scale bar: 200 μm.
(TIF)

S10 Fig *S*. *mansoni* developed *in vivo* for 28 days. Amplification: 100x. Scale bar: 200 μm.
(TIF)
