# Supplementary figures and images for: Validation of a human-serum-based in vitro growth method for drug screening on juvenile development stages of Schistosoma mansoni
Source: PLoS Negl Trop Dis. 2021 Mar 30;15(3):e0009313. doi: 10.1371/journal.pntd.0009313 (PMC8034724; doi:10.1371/journal.pntd.0009313)

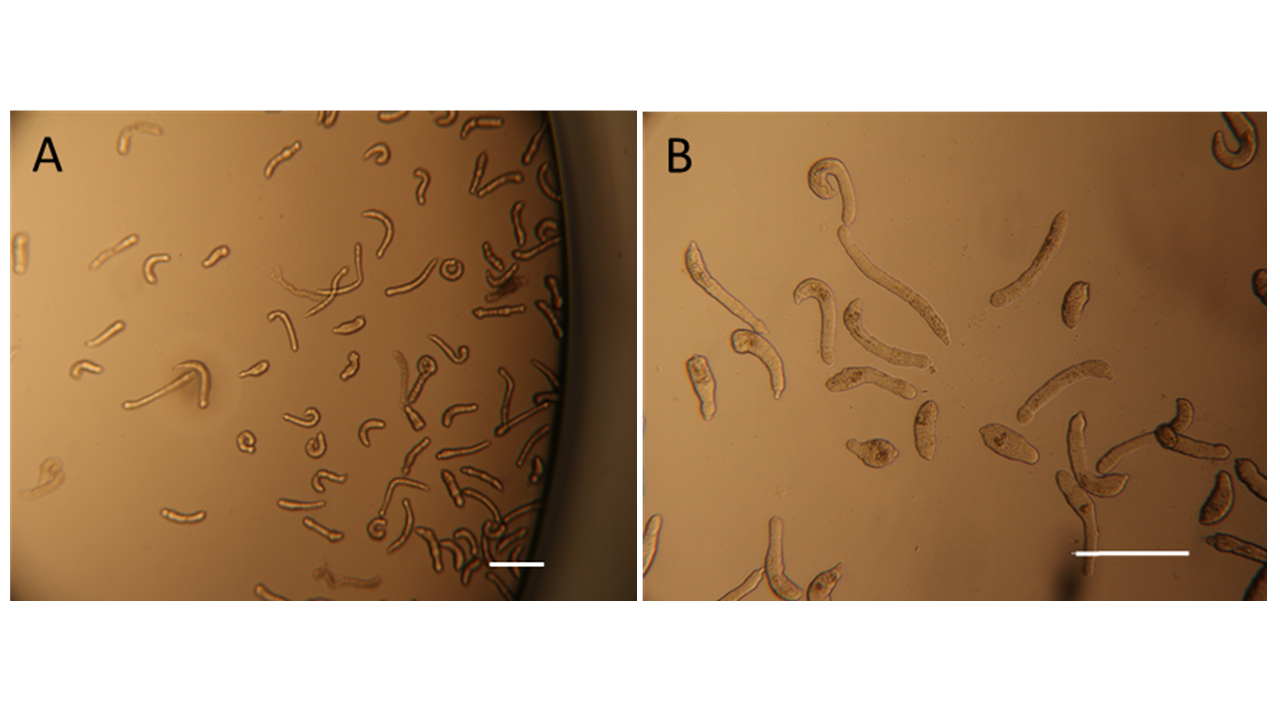

Supplement: S1 Fig — (TIF) [file pntd.0009313.s002.tif]

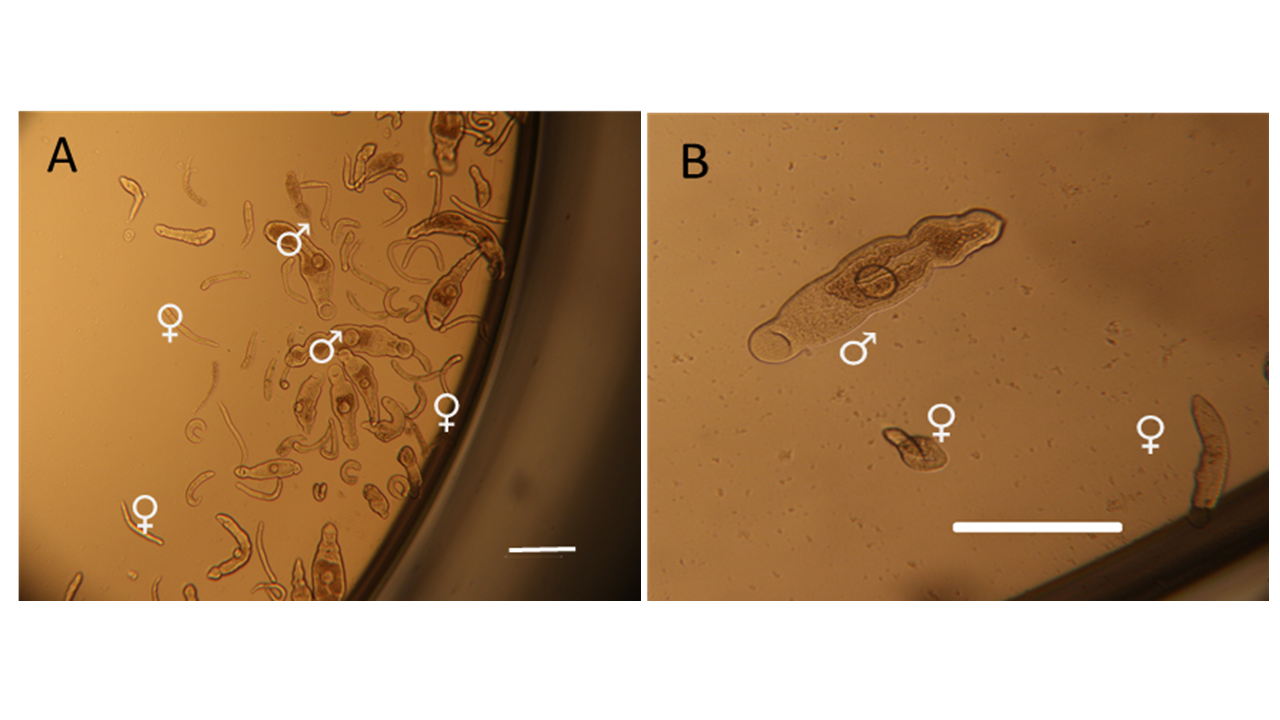

Supplement: S2 Fig — Amplification: A: 100x, B:200x. Scale bar: 200 μm. (TIF) [file pntd.0009313.s003.tif]

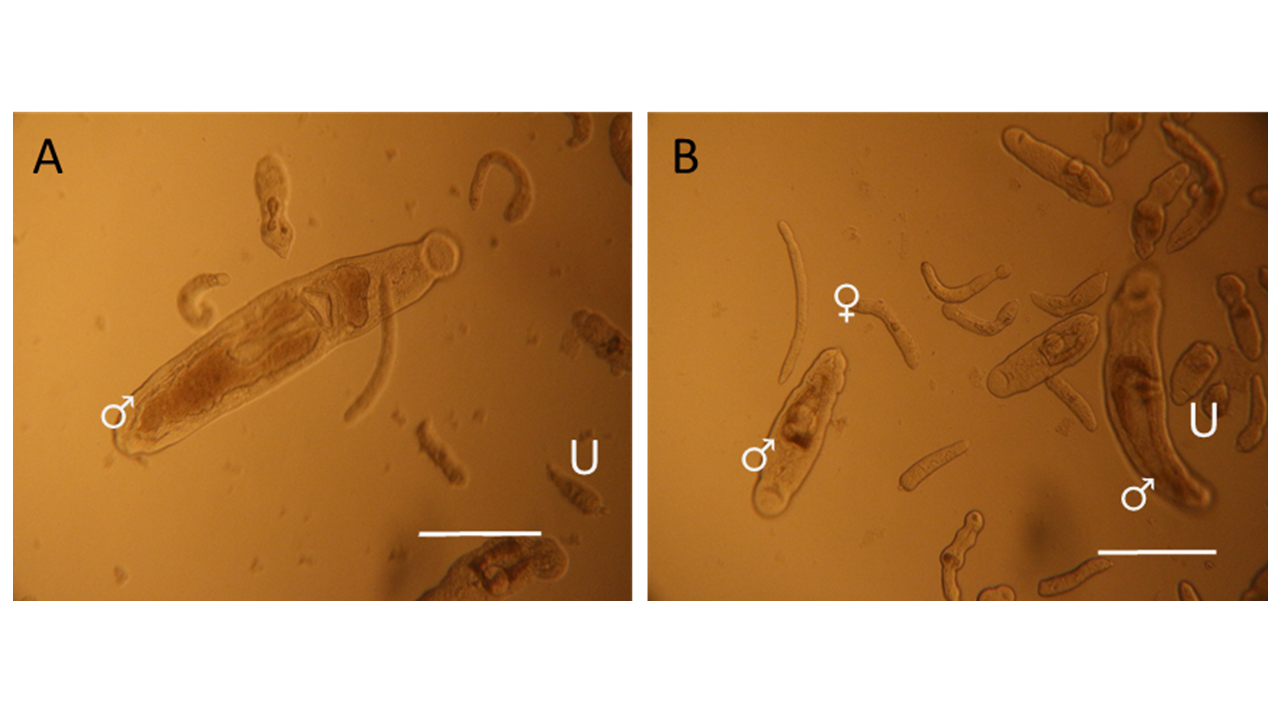

Supplement: S3 Fig — Scale bar: 200 μm. (TIF) [file pntd.0009313.s004.tif]

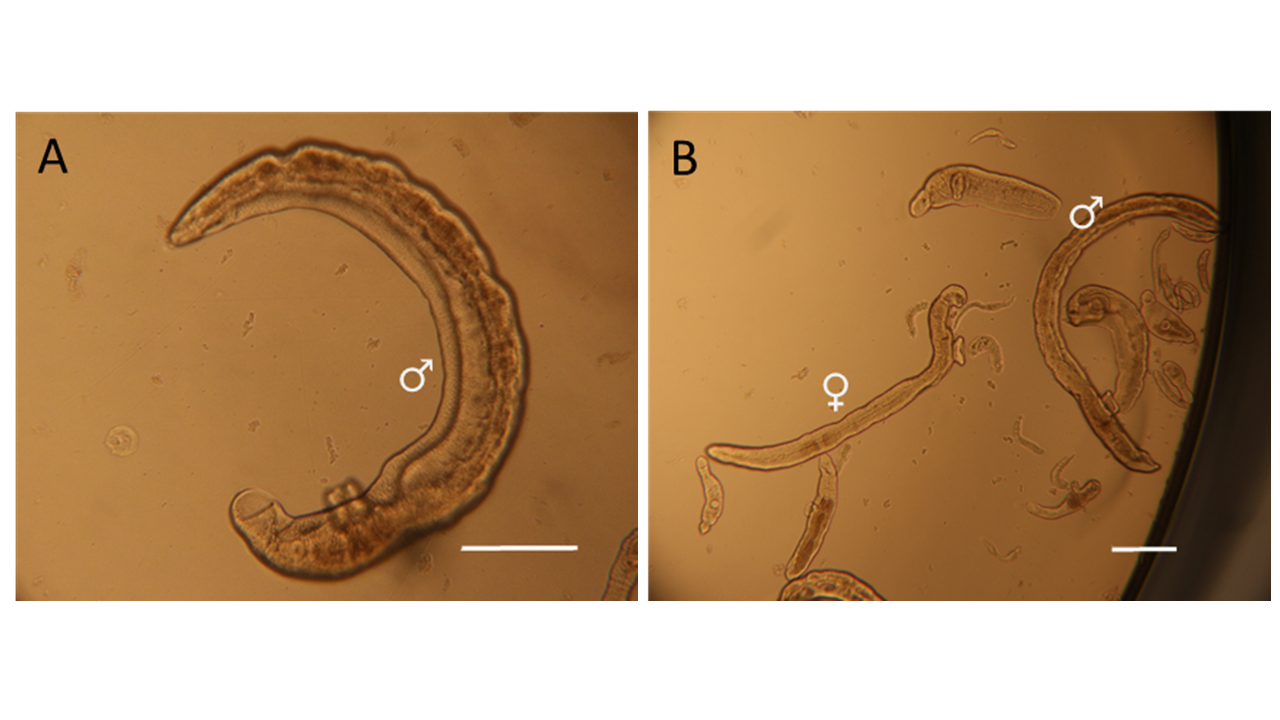

Supplement: S4 Fig — Amplification: A: 200x, B: 100x. Scale bar: 200 μm. (TIF) [file pntd.0009313.s005.tif]

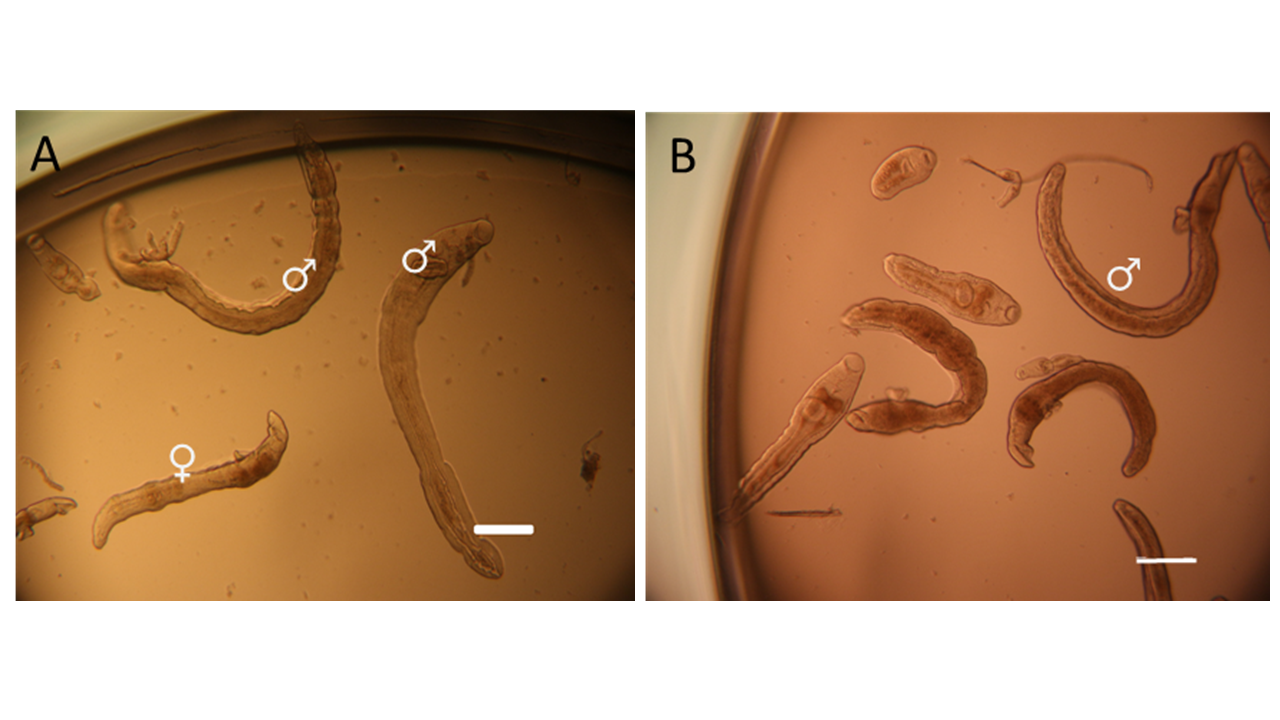

Supplement: S5 Fig — Scale bar: 200 μm. (TIF) [file pntd.0009313.s006.tif]

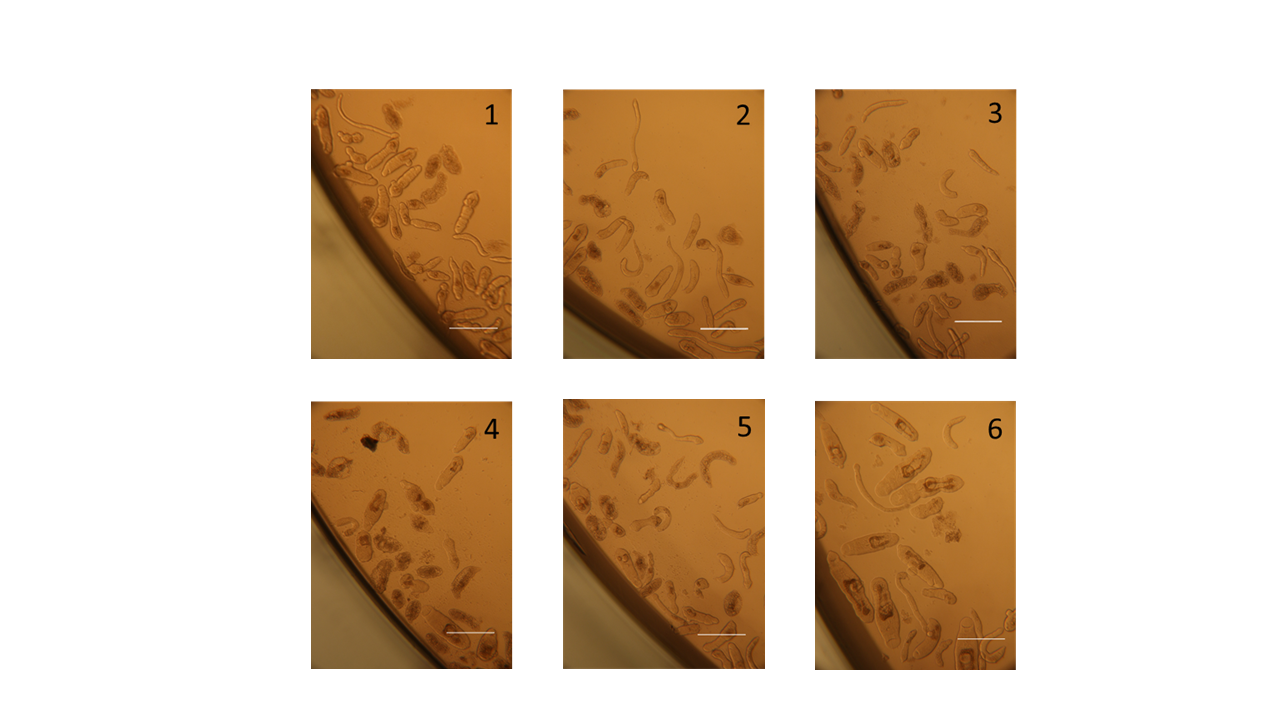

Supplement: S6 Fig — Comparison of the development of in vitro grown S. mansoni using HM + 20% HSe from five different donors by 21 days of development. Serum 6 is the combination of the other five sera mixed together in equal parts. Scale bar: 200 μm. (TIF) [file pntd.0009313.s007.tif]

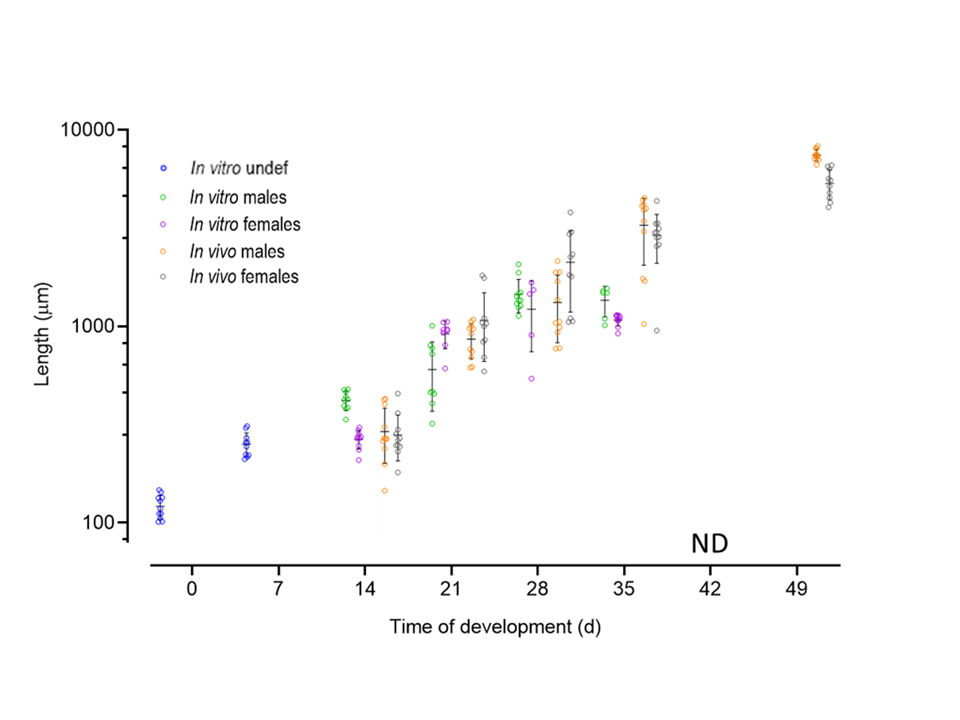

Supplement: S7 Fig — By means of either growing technique the worms’ gender is only to be identified after 10–13 days of development. Each point represents one worm. Abbreviations: ND: not done, undef: undefined: the gender of the worms cannot be determined by optical microscopy. (TIF) [file pntd.0009313.s008.tif]

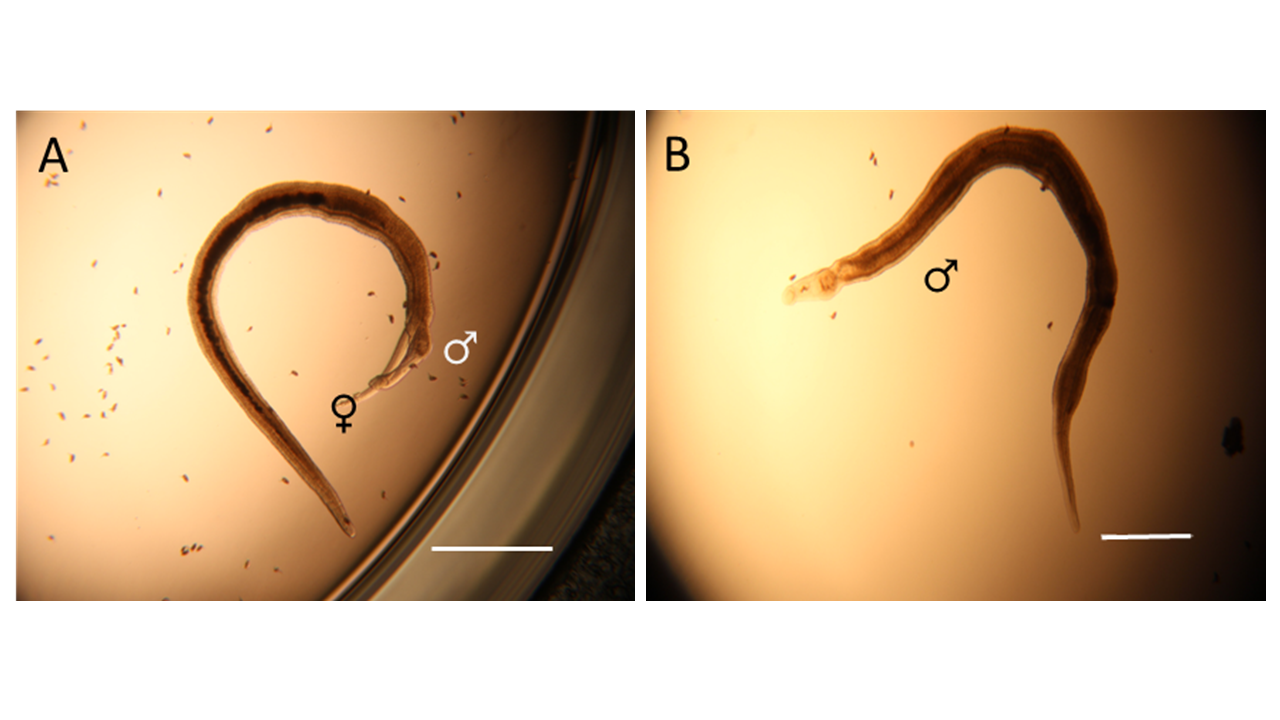

Supplement: S8 Fig — Amplification: 40x. Scale bar: 1 mm. (TIF) [file pntd.0009313.s009.tif]

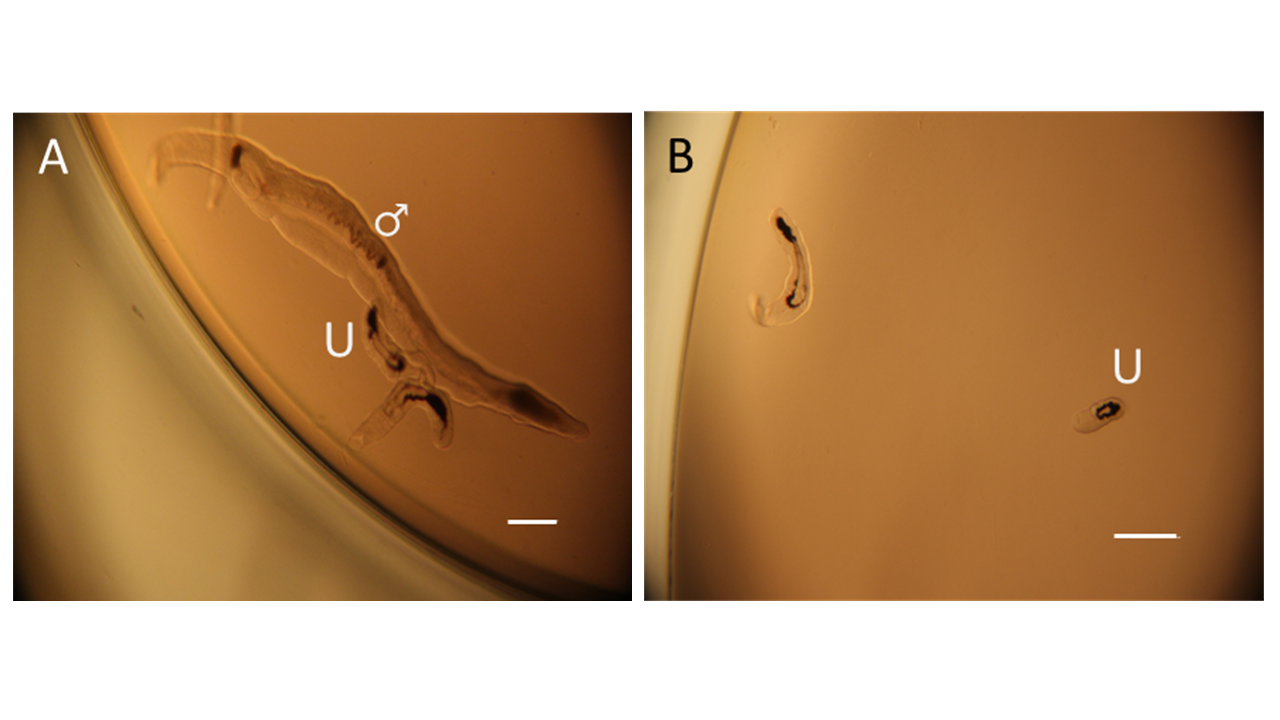

Supplement: S9 Fig — U: undeveloped worm. Scale bar: 200 μm. (TIF) [file pntd.0009313.s010.tif]

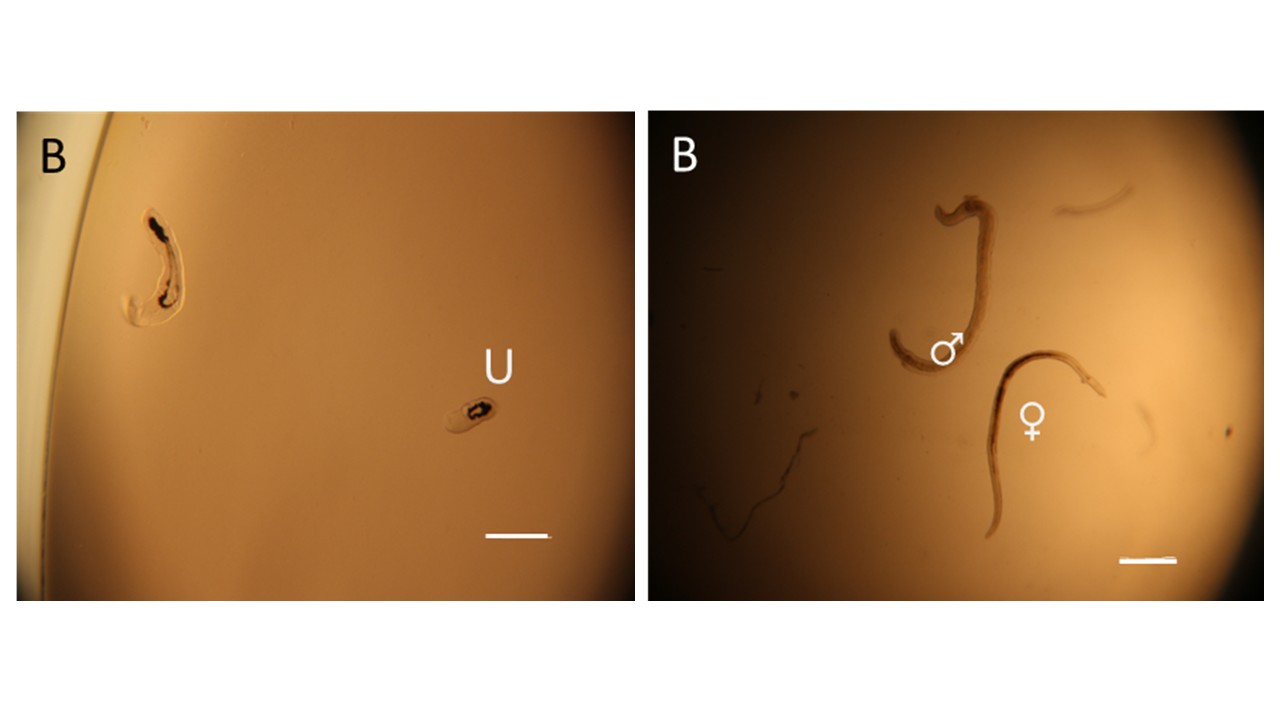

Supplement: S10 Fig — Scale bar: 200 μm. (TIF) [file pntd.0009313.s011.tif]
